# Uro-Genital Tuberculosis1The address on surgery, delivered before the South Carolina Medical Association, April 21, 1909, at Summerville, S. C.

**Published:** 1909-07

**Authors:** Bransford Lewis

**Affiliations:** St. Louis


### BUFFALO MEDICAL JOURNAL.

VOL. LXIV. JULY, 1909. NO. 12

### ORIGINAL COMMUNICATIONS.

### Uro-Genital Tuberculosis[1]

**With especial consideration of Tuberculosis of the Bladder**

BY BRANSFORD LEWIS, M. D., St. Louis.

IN acknowledging my deep appreciation of the honor you have conferred in inviting me to be your guest and essayist this evening, I am expressing the thought that is first and foremost in my mind. I have long looked forward to the time when I could have the opportunity of visiting this mother state, of which we hear so much in our part of the country,—and always in words of deep affection,—by your numerous sons who have been lost, strayed or stolen in that direction.

Though never before a visitor in South Carolina, my own Southern blood permitted not the slightest misgiving as to whether I should feel "at home" on arrival here; I only looked forward to the pleasure that I felt was in store for me; of coming face to face with the scenes and birthplace of a large share of American history; of events that thrill the loyal heart of every American citizen. I wanted really to see Fort Moultrie, where Sergeant Jasper replaced the flag, and to become personally acquainted with your metropolis, Charleston, that has occupied the lime light in so many eventful incidents, from cannon balls to earthquakes and, anon, from pills to politics.

But, coming down to the subject that is to engage our attention just now, an easier and clearer conception of uro-genital tuberculosis is obtained, when the general scheme or plan of attack made on these organs by this infection is understood. It is claimed by certain students of this subject that tuberculosis is never primary in the urinary or sexual organs; that, although its first *clinical manifestation* may be focussed here, the development is really secondary to some unrecognised involvement elsewhere,—a latent pulmonary lesion, or even a lymph gland.

1. The address on surgery, delivered before the South Carolina Medical Association, April 21, 1909, at Summerville, S. C.

However this may be, there are certain general plans followed in the development of the infection when it does involve the genitourinary system, that now seem fairly well understood and attested.

Transmitted in any one of five different ways, the tubercle bacilli reach the genitourinary organs (1) through the blood-vessels; (2) through the lymph channels; (3) by means of the physiological secretions, the urine, semen; (4) by continuity of tissue; (5) by contiguity.

Being thus transmitted, it is found that certain organs of the genitourinary system habitually receive the brunt of the attack first, while others invariably stand secondary in this respect. In the vast majority of all cases in which the uro-genital organs are implicated, one or the other kidney is the location of the first attack (never both at the same time); in a very small proportion of such cases one or the other epididymis is the point of initial attack.

From these respective foci extension of the infection spreads in the direction of the physiological secretions (urine and semen); that is, from the kidney downward, along the ureter to the bladder, and from the epididymis upward, along the vas deferens, the seminal vesical ejaculatory duct, prostate to the urethra and vesical neck. Thus the bladder stands in the line of attack from either direction; and therefore stands a most excellent chance of being ultimately concerned in the disease process, no matter from whence it comes.

The determination of these facts (chiefly contributed by Motz and Hallé, *Annales des maladies des organes génito-urinaires*, 1906, XXIV., p. 161) has finally settled the old time discussion on "ascending and descending infection," as to whether the infection ascended from bladder to kidney, or descended from kidney to bladder. In the presence of infection of both kidney and bladder, it is assured that the kidney is the organ primarily involved, and the extension has been to the bladder, either by the ureter or its lymph ducts. If the infection has come up from the genital gland, the bladder stands in the same secondary relationship also.

In other words, *vesical tuberculosis is an affection invariably secondary to tuberculosis of some other uro-genital organ*, and must be so considered in respect of diagnosis, prognosis, therapy and management.

Probably we would have difficulty in getting a patient affected with tuberculosis of the bladder to acknowledge that the bladder was "secondary" in any respect. It is usually the most absorbing and vivid exemplification of misery incarnate that he can imagine.

*Location of Early Evidences of Vesical Tuberculosis.*—The early expression and location of the disease are much influenced by the mode of production of the infection. If the implantation is an ascending one, from testes and vesicles or prostate, the early manifestations will be on the trigone or the vesical neck; but if the germs have descended from a kidney by way of a ureter, the ureter itself or its immediate neighborhood is the point that first capitulates and becomes reddened, swelled, inflamed and finally ulcerated.

*Marital Tuberculosis.*—It has been claimed that sexual intercourse presents a mode of direct transference of tubercle bacilli and infection that shows itself with sufficient frequency to be taken into account. I have myself observed instances of this sort, one in particular in which tuberculosis of the bladder occurred in a wife whose husband, a physician, was the subject of advanced general tuberculosis, and the coincidence was very striking, but investigation into the history of the wife showed that her own family history was not above suspicion. This question has been the subject of careful scrutiny and analysis by Mr. E. G.Pope, of the Adirondack Sanitarium, who concludes that there are too many sources of error as yet to fix the responsibility of direct contagion on matrimonial association. He believes, further, that assortive mating (the tendency among subjects of certain diseases to select in marriage those with like marked tendencies as themselves) accounts for two-thirds and infective action for not more than one-third of the whole correlation observed in these cases (quoted in *Journal of American Medical Assn.*, September 5, 1908). If this mode of infection were a frequent factor the woman would be the chief sufferer, from the deposition and retention of tubercle bacilli in the recesses and folds of the vagina, but records indicate that women are affected with vesical tuberculosis about one-third less than men.

*Mode of Development.*—The stages of development of tuberculous cystitis may be divided as follows (Motz and Hallé):

1. The stage of invasion and formation of tubercles.
2. The stage of inflammation and superficial ulceration.
3. The stage of deeper infiltration.
4. Stage of widespread destruction (Walker).

In the earliest period (invasion) there are white or grey tubercles and injected areas, without the presence, necessarily, of ulceration. Some have sought to call this "tuberculosis of the bladder" as differentiated from tuberculous cystitis, but the differentiation is artificial; they are stages of the same process.

Ulceration shows itself after the apex of the tubercle becomes necrotic and breaks down. These independent ulcers tend to coalesce and make the larger ulcers that are typical of the process.

Such ulcers are sharp-edged, sometimes slightly undermined, with their surface irregular and uneven, and liable to be covered with greyish pseudo-membrane, tinged with blood or small clots, at times. The ulcer is surrounded by membrane whose natural luster is dimmed, which looks reddened and velvety, and whose bloodvessels are lost in the intense injection prevailing.

*Clinical Evidences.*—The clinical evidences may be described as those giving rise to (a) suspicion; (b) confirmation; (c) conviction. Suspicion of vesical tuberculosis should be aroused by persistent and apparently inexplicable frequency of urination, either by night or day or both, together with the appearance, over long periods of time, of blood-cells in microscopic quantities, in the urine. In the later stages pain and harassing suffering, day and night, with interruptions or loss of sleep and lowering of nerve stamina, mark the more serious phases of the disease. Persistent or recurrent bleeding from the ulcerated areas is apt to take place, adding to both, mental and physical depression. But of much more importance, from the diagnostic standpoint than this, is the persistent appearance in the urine of red cells *in microscopic quantities* in the *early periods* of the infection. It is important *because it is early,* furnishing the medical attendant the grounds for suspicion that, if recognised and followed up, enables him to recognise the disease at the earliest possible moment, which is, of course, all-important. But I have found in numerous instances that this feature was not recognised or even suspected because examinations of the urine were not sufficiently careful or searching. Such urines are often limpid clear, and from the microscopical standpoint as far above suspicion as Cesar's wife could have been; yet, when searched after prompt and thorough sedimentation are found to contain large numbers of red cells, present in every specimen voided. Frank hermaturia may become one of the outspoken danger signals later, when inflammation or ulceration is established.

Pain is either spontaneous or excited by the act of urination. In the latter case it is most intense at the end of the act, aroused by the squeezing of the inflamed and ulcerated areas by the vesical sphincters. Pain of this character is almost a constant attendant, at one period or another, on the tuberculous bladder, and often becomes so severe as to precipitate the patient into cocaine or morphine addiction. But while this is true, another allied symptom is even more characteristic of the disease, that in itself alone often elicits the suspicion of tuberculosis: that is, excessive tenderness and hypersenitiveness to manipulation. Appreciation ing, though possibly the right carotid might have been tied as of this fact should lead to the free use of local anesthesia for making any local investigation.

In health the desire to urinate is aroused by irritation of the mucous membrane of the prostate, urethra, or neck of the bladder. Any irritation applied at this point will arouse desire to urinate. When that irritant is urine from the full or over-filled bladder, the result is physiological desire to urinate, that passes off when the bladder is evacuated; when the irritant is tuberculous inflammation or ulceration, there is good reason for the persistent and intense desire to urinate that so frequently accompanies this condition, even in the presence of a comparatively empty bladder. This fact is contributed to by reason of the frequency with which the neck of the bladder is the part especially involved. Sometimes the frequency is due to reflected irritation onto the neck, rather than direct inflammation there, as for instance from renal tuberculosis, which occasionally causes frequency before the involvement of the bladder.

Among the characteristic signs of vesical tuberculosis is a marked diminution in the capacity of the bladder (contracted bladder). From three to four ounces is a fair average capacity for such bladders; due to infiltration and thickening and lessened elasticity of the walls.

A peculiarity noticeable is that in the face of this condition and the prolonged inflammation prevailing with it, the urine is liable to be acid in reaction. The introduction of the proteus group of bacteria sets up ammoniacal fermentation that alters this, however, establishing alkalinity and adding to the subjective complaints and activity of inflammatory processes.

*Mixed Infections.*—In addition to the tubercle bacilli found in tuberculous cystitis, other organisms are sometimes present and take an active part in the pathological processes. Some of them have something to do with the inauguration of the tuberculous infection, while others are followers of the tuberculous infection, adding their quota to the miserable conditions prevailing. Many cases of urinary tuberculosis have been observed as followers of urethral gonorrhea; while streptococci, staphylococci, colon bacilli and other organisms have been found as companions of tubercle bacilli. It is considered probable that pyogenic organisms lower the resistance of the mucosa and produce minute breaks in its surface, allowing the tubercle bacilli to enter the submucosa. On the other hand, it is thought that cystitis caused by members of the proteus group does not offer so fertile a field for the invasion of tubercle bacilli as that produced by streptococci and gonococci (Walker).

*Diagnosis.*—While the characteristic symptoms and signs given may be sufficient to give rise to suspicion and even confirmation of the suspicion of tuberculosis cystitis, in other words, a presumptive diagnosis of that affection, the crucial factor for determining the diagnosis, as with tuberculosis of other parts of the body, is the demonstration of the tubercle bacillus in connection with the convincing evidences of inflammation of the bladder. The appearance of tubercle bacilli in the urine does not by any means necessarily indicate vesical tuberculosis, as it has repeatedly been proven (Israel, Jani and Nakarai, Thilicwicz) that tubercle bacilli may float in the urine of persons whose urinary tract is innocent of any pathological lesion, even as demonstrated post mortem.

The finding or identification of the tubercle bacilli in the urine is not always easy or possible and is largely dependent on accompanying conditions, the age and extent of the lesions, the mode of search carried out, and the like. The bacilli may be sparse in number and on that account escape detection by the ordinary methods. Bryson called attention to a useful procedure in this connection—namely, the draining off by sterile catheter of the small amount of urine left over after voluntary urination, as offering a better probability of gathering the bacilli that have settled at the bottom of the ladder.

*Differentiation Between Smegma and Tubercle Bacilli.*—Much thought and research have been spent in the endeavor to find a method of definite differentiation by the microscope between the smegma and the tubercle bacillus. Many different staining methods have been evolved. I have seen some serious errors result from relying on such methods. I believe it is much safer and better to pay no attention to them whatever, but to make the differentiation by excluding the introduction of smegma bacilli in obtaining the specimen. It should always, where possible, be obtained by sterile catheterisation, in either male or female, after thorough cleansing of the external genitals. In the female one must be especially careful even then, as the urethra is short enough to permit of the introduction of smegma bacilli on the catheter as passed into the bladder.

Sedimentation should be effected promptly after the passage of the specimen, and should be thorough. Trevithick recommends that the supernatant fluid be poured off from the first sediment obtained, and the tube be refilled with water and resedimented. This is supposed to favor the adhering of the bacilli to the glass slide or cover-slip. A number of staining methods are of value, the carbol-fuchsin retaining the esteem in which it has long been held.

Failure to find bacilli in the specimen, even after several essays, does not prove their absence, and merely stands as negative evidence; it must be supplemented with the more accurate mode of guinea-pig inoculation of the urinary sediment. This is reliable and extremely valuable—much more so, in the writer's opinion, than the hypodermic tuberculin test. But it requires from two to three weeks' time for maturing.

*Cystoscopy.*—The other necessary factor for fixing the diagnosis of tuberculous cystitis is the picture presented by the cystoscope.

The writer desires to express himself as decidedly opposed to the rather broadly disseminated view that urinary tuberculosis means interdiction of the use of instruments, either for diagnosis or for treatment. While due conservatism should be exercised in this regard, the idea that it is a forbidden field should be abandoned. That idea is no more applicable here than in other conditions requiring the ministrations of surgery and medicine. It has been observed in a number of cases of severe urinary tuberculosis that the repeated use of the cystoscope, together with ureter catheterisation and other forms of instrumentation, have not retarded or interfered with progressive improvement or even recovery. In some instances, indeed, such measures themselves were followed by distinct improvement, sustained in repeating them. I have never yet observed any complications or serious consequences from such manipulations, though they are restricted always to the necessities of the case and not used in a meddlesome way.

The writer has been much gratified to observe participation in this view by Willy Myer (*New York Medical Journal,* April 27, 1907), who says:

Cystoscopy of the urinary tract has always been a subject of great interest to me, and I look upon it as a valuable diagnostic aid. I was somewhat surprised, therefore, to note the opinion expressed by a few colleagues that this examination as well as catheterisation of the ureters is contraindicated in patients afflicted with this trouble (tuberculosis). In all my cystoscopic experience, extending over a period of just twenty years, I have never seen cystoscopy cause direct detriment to these patients, and I feel it never should, so long as the instruments are gently and judiciously manipulated. I would therefore state right here that I consider cystoscopy not only permissible but in most of these cases absolutely necessary, in order to enable us to establish a definite diagnosis.

While characteristic lesions, such as have previously been mentioned, are shown by the cystoscope in tuberculous inflammation and more especially ulceration of the bladder, it can not be said that the lesions are always typical, or that they adhere to hard and fast lines of development. On this subject Casper (Bonney's translation, p. 224) says:

In general, tuberculosis of the bladder does not present a specific picture. Besides diffuse swelling and redness there are at times deeply congested localised areas clearly separated from apparently healthy tissue, while again ulcerations having nothing distinctive about them are seen. Tubercles are very seldom found.

Fenwick (Clinical Cystoscopy, page 172) says:

The primary (early) deposit is detected on the posterior wall in two forms, either diffuse, as a dull red patch or patches, or localised, as a single ulcer to the inner side of the ureteric orifice. The dull red patches betoken extravasation and exudation. I have sometimes met with cases in which a solitary ulcer was seen to the inner side of the orifice and I could not distinguish its appearance from the solitary simple ulcer.

*Ureteral Orifices.*—Besides the oval or rounded ulcers with sharp edges, perhaps undermined, with roughened, raw and bleeding surfaces, surrounded by a zone of congestion, of velvety appearance, lacking the natural luster, with other areas of congestion either localised or diffuse—besides these characteristic appearances of the bladder mucosa itself, the ureteral orifices themselves ordinarily but not invariably present characteristic features in connection with descending tuberculosis. These have been especially well studied by Fenwick, who says (ibid., p. 174): "One of the ureteric orifices is attacked before the other; both are never attacked equally. The orifice of that ureter on which the stress of the disease first falls changes in contour, its lips thicken, and it becomes caked and patulous. The same changes will be found in the corresponding renal pelvis, with implication of the lower part of the kidney." The same author says that where the vesical implication is a descending one from a tuberculous kidney that retracts under the ribs, the ureter becomes stretched (tense), the vesical orifice of the ureter becomes *displaced,* elevating the trigonal angle of that side and perhaps presenting a funneled appearance there—furnishing a diagnostic sign of material value. Instead of being an inch and a half from its fellow and the same distance from the urethral outlet, it is found to be as much as two inches from either opening, and is drawn both outwards and upwards.

While the positive evidence thus presented by the appearance of the ureteral orifice should be given its full meed of credit as an indicator of the condition of the kidney above it many authors go still further and accept its negative evidence with as great confidence. They declare that if the ureteral orifice is normal in appearance there is no necessity of looking further for evidence of tuberculosis of the corresponding kidney; they give it a clean bill of health on that ground alone.

Others, again, accept with equal complacency the cystoscopic appearance of the urine as it is being emitted in jets from the respective ureteral openings, saying that if either jet is cloudy or bloody it means that the corresponding kidney is the one at fault. The acceptance of such flimsy and untenable evidence can only result in discrediting and defaming a procedure of the highest scientific value. Kidneys saturated with tuberculous infection have been removed from patients whose ureteral orifices showed no indication whatever of the renal trouble, and if it be true that urine macroscopically clear and to casual inspection healthy, can contain pus cells, red blood cells and tubercle bacilli, the cystoscopist must be expert, indeed, to detect these in the jets of urine issuing from the ureters.

The reliable, safe and secure mode of arriving at the determination of implication or health of the respective kidneys is that by ureteral catheterisation and minute investigation of the urines drained thereby. There are no well grounded objections to the procedure, and the information it furnishes is not illusory or misleading. Much time and printer's ink have been spent in the endeavor to prove that ureteral catheterisation is dangerous because of its liability to convey infection into a healthy ureter. If this objection were tenable it would have had plenty of evidence ere this to justify it, but such has not been the case; and of the many thousands of ureteral catheterisations, none have been reported as leaving such a heritage.

Segregation does not compare with it in any respect. It is both unreliable and inefficacious, leaving no material reason for its use.

*Lymphoid Tubercle.*—A peculiar formation occasionally seen in the bladder either in the presence or absence of tuberculous infection, is a deposit of lymph in the form of lymphoid tubercle. These little tubercles, yellow and prominent, dot the surface of the vesical membrane in a manner that is startlingly like the tubercles from Koch's organism, but with which they may have no relation. Alexander describes the condition under the term, nodular cystitis. Kneise in his Handatlas der Cystoskopie, 1908, Table V., No. 22, gives a beautiful illustration of the condition, which shows very well also the chief differentiating features betwen these and real tubercles. there is absence of the zone of inflammation and extravasation that habitually accompanies true tubercle. In a case observed post mortem by Dr. Mark and Dr. Hall, of Kansas City, the pseudo-tubercles were scattered over the entire bladder mucosa, but this is exceptional, as they ordinarily affect only the trigonal neighborhood.

It is evident, therefore, in connection with cystoscopy that while seeing is believing, and the cystoscopic picture is essential and all-important, it must be correctly seen, appreciated and interpreted; and conclusions from it should be made only after due consideration of the clinical history and general features of the case.

The "therapeutic test" is a means which, applied either inadvertently or intentionally, gives a fairly good indication of the tuberculous or nontuberculous nature of a vesical inflammation. Nitrate of silver solution, nearly always gratefully received by the infected bladder, arouses prompt additional irritation if the cystitis is of tuberculous origin.

The tuberculin test of inoculation should have a place here as elsewhere in bearing testimony on the presence or absence of tuberculous infection.

The final definite diagnosis must after all occasionally be made without the demonstration of the tubercle bacillus and in spite of indefinite cystoscopic findings, the other indications present being taken as sufficient to justify a conclusion. One sometimes *feels* that he is in the presence of tuberculosis, without being able to elicit the distinctive, individual evidences desired. Then, too, the condition of other organs of the body, whether tuberculous or not, may have a serviceable bearing on the question.

*Treatment.*—General considerations. The treatment of tuberculous cystitis may be divided into palliative and curative: by means of hygienic, systemic, local and operative measures.

Since this affection is a secondary one, with the original focus situated at some other part of the body—in most cases the kidney—the *curative* plan usually involves an operative attack with removal of the original focus.

The modes of treatment may be divided as follows:

1. General, systemic and hygienic measures.

2. Tuberculin treatments.

3. Operative attack on the original foci of the infection.

4. Operative attack on the bladder: (a) extirpation of the bladder and transplantation of the ureters; (b) curetting or cauterisation, or both; (c) local application of bichloride or carbolic solution; (d) drainage of the bladder by fistula.

5. Local applications to the bladder: boric acid irrigations, bichloride instillations, injection of iodoform oil or other soothing, antiseptic medicaments.

6 Cystoscopy and ureteral catheterisation and application of medicaments through them into the ureters and renal pelves.

*General Hygienic Measures.*—The various systemic and hygienic influences suitable for tuberculosis in general are indicated, and it would be supererogation to rehearse them here. While in entire harmony with the belief that these are of prime importance and must be supplied in each instance, I do not participate in the belief that they must be attained at all hazards; in other words, I am of opinion that so much good can often be accomplished by local operative measures that these should not be sacrificed merely for the purpose of going away for "good air" and ideal hygienic influences. This is in opposition to the doctrines of certain writers, I know, but experience has brought me to that conclusion.

Creasote and guaiacol in full dosage are often beneficial; likewise various systemic tonics that are nonalcoholic. The formaldehyde group of medicaments (urotropin, cystogen) is contraindicated; they afford no service and are often highly irritating.

*Tuberculin Treatment.*—Tuberculin treatment occupies the same position here that it does with reference to tuberculosis of other parts of the body, and should be prescribed under the same regulations. The dosage should be minute and short of producing the marked and injurious reactions prevalent in the earlier use of this remedy.

*Operative Treatment.*—Operative attacks embrace: (a) nephrotomy and partial resection of involved kidney tissue; (b) nephrectomy; (c) ureterectomy; (d) castration or epididymectomy; (e) vasectomy; prostatectomy; (f) removal of seminal vesicles.

Partial resection of a tuberculous kidney has proved a false hope and has been abandoned. It does not secure the removal of all of the original focus.

Experience in manifold instances has proved the justification of removing the kidney, if it be the originating factor, with possibly the corresponding ureter if seriously involved. This is also true of the removal of the worse of two tuberculous kidneys, in certain instances, relieving the sufferer of a suppurating and infected organ that is doing no good but is undermining the health and inciting infection elsewhere. It is well established that bladders the subject of tuberculous inflammation and ulceration are reclaimed from their unhealthy condition by these measures and undergo definite reparative changes, the ulcers heal and inflammation ceases.

Certain authors lay much stress on the removal of the entire ureter, in addition to the kidney, where it is affected, insisting that if any of it be left behind it will necessarily prove a source of renewed infection later. This latter claim has not, however, been established. As already mentioned, the tuberculous bladder is able to clear up and regain its health after removal of the primary focus, a kidney; why not the ureter as well? While it can not be denied that it is theoretically better to be rid of a tuberculous ureter, its removal in very serious cases cannot fail to add to the duration and perhaps difficulty of the operation, possibly to the extent of compromising the chances of the patient's going through it successfully. In view of the doubtful advantage gained is it advisable to run the risk in all instances? I confess my own leaning to the negative side of the question. Two years ago, acting on this reasoning, I removed the left kidney of a young woman who was so debilitated in health and strength that the several medical attendants were united in the belief that she could not withstand the combined operation of nephro-ureterectomy. The ureter was left behind; the patient, though in a precarious condition for several days, recovered. Her bladder has since been relieved of both inflammation and ulceration; the ureter of that side has become obliterated, discharges nothing and is not even patent to a catheter. The patient has gained about thirty pounds and her general health appears restored. For a time after the operation there was tenderness along the line of the affected ureter, but that passed away, giving no further trouble.

Graphically portraying the favorable progress that has been made in the past twenty years by improved surgical technic and more prompt and definite diagnosis, the estimates of mortality of nephrectomy for tuberculosis as given by the following several writers and operators at different periods may be cited:

In 1890 by Brodeur, 58 per cent mortality; 1893, Palet, 40 per cent.; 1902, Pousson, 29.4 per cent.; 1902, Schmieden, 31.3 per cent.; 1905, P. Wagner, 24.5 per cent.; 1907, Blum, 13.3 per cent.: 1908, Blum, 11.1 per cent.

The same author (Blum, loc. cit.) has formulated the contra-indications to nephrectomy to the following effect: (1) unduly advanced age of the patient; (2) the presence in the patient of general miliary tuberculosis; (3) the co-existence of malignant diseases of other organs, of diabetes of serious degree; (4) advanced pulmonary tuberculosis that offers little hope of recovery; (5) widespread tuberculosis of the genitals with the bladder included.

*Counterindications.*—The justification for operating on or removing the testes, epididymes, vasa deferentia, vesicles or prostate, when they are original foci, seems to be not yet established for all cases, and there is no such uniform sentiment here as there is with respect to the removal of the kidney. So much can be accomplished by general and hygienic measures that it would seem desirable to first adopt these in such cases, and defer any mode of operating until its necessity is demonstrated by persistence or rapidity of development of and extension to other organs. Factors present in individual cases will have much to do with determination of the question.

Operative attacks on the bladder, except for palliative purposes (for necessary artificial drainage, etc.) are, on the whole, illogical and have little to recommend them. The secondary nature of the affection explains this fact.

*Local Applications and Treatments.*—Any local measures adopted should be as little irritating as possible, and while instrumentation should be reduced to a minimum, that does not preclude the use of the catheter or the cystoscope when they are needed. Obstructive conditions of the urethra should be remedied, securing free and easy transit for the urine, for which dilating methods are preferable to cutting.

Irrigation of the urethra and bladder are often serviceable where there is secondary infection. Much has been claimed for bichloride instillations, by the French school particularly, and Rovsing has lauded the effect of introducing strong solutions (5 per cent.) of carbolic acid into the bladder, but my personal experience and the reports of others are adverse to such heroic, irritating methods. The most serviceable local application I have used is oil emulsion of iodoform, say, a dram to the ounce of some bland oil, such as liquid vaselin. It is injected once daily, after emptying the bladder, and the patient is instructed to retain it as long as practicable, not expelling the "last drops" in urinating. The writer has used the same emulsion as an injection into kidney pelves affected with tuberculosis, and apparently with much benefit, in several cases. A male patient who has been under observation for four years and was formerly severely afflicted with the various effects of vesical and left renal implicaplication, has never been subjected to operation but has devotedly carried out all hygienic and local measures advised. He at first received a number of injections of the iodoform oil into the pelvis through the ureteral catheter, and has himself used the vesical injections of the same. In the period mentioned he has not only been relieved of all of the acute symptoms, the pain and frequency of urination and the like, but has regained much weight, strength and faculty for active employment. Test of his urine by guinea-pig inoculation recently failed to disclose any evidence of the tuberculous infection.

Instances of improvement or conditional recovery under nonoperative measures are not unique in my experience. They are of sufficient number to give rise to a serious question as to whether all such patients should necessarily be subjected to operation on discovery of a moderate nidus of infection in one kidney, with possibly beginning of involvement of the corresponding ureter or the bladder. I believe I should rather make haste slowly and first try the effects of the local and hygienic measures to demonstrate the extent of their benefit. Meantime the patient will probably be brought to a better condition for operation if it prove necessary than he was before the institution of those measures. Where there is serious involvement of a kidney, the status is entirely changed and the sooner it is removed the better.

*Palliative Operations.*—In some instances the pain and irritation from tuberculous inflammation are so excruciating that some operative plan has to be adopted to alleviate the distress, if nothing more. The one usually favored is that of establishing a suprapubic fistula, permitting continuous drainage of the bladder into a rubber receptacle which covers the opening. To the same end, Simpson suggested resection of the sensory nerves that come off from the third and fourth sacral trunks, and Watson has advised double lumbar nephrostomy, for the purpose of draining the urine directly from each kidney and not permitting its descent into the bladder.

1050 Century Building.

